# Prediction of Voice Fundamental Frequency and Intensity from Surface Electromyographic Signals of the Face and Neck

**DOI:** 10.3390/vibration5040041

**Published:** 2022-10-13

**Authors:** Jennifer M. Vojtech, Claire L. Mitchell, Laura Raiff, Joshua C. Kline, Gianluca De Luca

**Affiliations:** 1 Delsys, Inc., Natick, MA 01760, USA; 2 Altec, Inc., Natick, MA 01760, USA; 3 Department of Biomedical Engineering, Boston University, Boston, MA 02215, USA

**Keywords:** pitch, loudness, EMG, voice, speech, fundamental frequency, intensity

## Abstract

Silent speech interfaces (SSIs) enable speech recognition and synthesis in the absence of an acoustic signal. Yet, the archetypal SSI fails to convey the expressive attributes of prosody such as pitch and loudness, leading to lexical ambiguities. The aim of this study was to determine the efficacy of using surface electromyography (sEMG) as an approach for predicting continuous acoustic estimates of prosody. Ten participants performed a series of vocal tasks including sustained vowels, phrases, and monologues while acoustic data was recorded simultaneously with sEMG activity from muscles of the face and neck. A battery of time-, frequency-, and cepstral-domain features extracted from the sEMG signals were used to train deep regression neural networks to predict fundamental frequency and intensity contours from the acoustic signals. We achieved an average accuracy of 0.01 ST and precision of 0.56 ST for the estimation of fundamental frequency, and an average accuracy of 0.21 dB SPL and precision of 3.25 dB SPL for the estimation of intensity. This work highlights the importance of using sEMG as an alternative means of detecting prosody and shows promise for improving SSIs in future development.

## 1. Introduction

Speech is the basis of human interaction. For many languages, spoken communication is not only governed by the words that make up a message, but also the relative emphasis of syllables within each word. Often conveyed by changes in prosody—including vocal characteristics of pitch, loudness, voice quality, and temporal variability—it is *how* the words are said that facilitates understanding, conveys meaning, and grants nuance to an interaction. Through unique modulations in these characteristics, individuals can develop their own speaking style and identity. However, people with a limited ability to produce speech, such as those who undergo laryngectomy due to trauma or disease, lack this natural method of self-expression. Consequentially, those affected often struggle with daily communication and tend to face psychosocial challenges, including difficulty integrating at work, social withdraw, depression, addiction, anxiety, and altered self-identity [1–5].

The development of assistive technologies known as silent speech interfaces (SSIs) attempts to bridge this gap in self-expression by providing an alternative method of communication that is independent of an acoustic signal. Instead, SSIs leverage other physiological signals to infer information about speech content and reconstruct this content as text or audible outputs [6]. Different approaches have included ultrasound and optical cameras [7–9], electropalatographic [10], or electromagnetic [11] devices for tracking tongue and lip movements; non-audible murmur microphones for detecting resonance in the vocal tract [12,13]; surface electromyography (sEMG) of articulatory muscles or the larynx (e.g., [14–18]); and motor cortex implants [19], electroencephalography [20] or electrocorticography (ECoG; [21]) to track speech-related brain activity.

Despite the advances in SSIs, the resulting synthesized speech often lacks prosody and, as a result, tends to sound monotone and unnatural. Recent work to overcome this shortcoming by Herff et al. [22] demonstrated that an SSI utilizing EcoG could preserve linguistic and conversational cues, wherein listeners found the synthesized speech to be intelligible 66% of the time. However, the system itself requires a craniotomy to operate, making it an invasive option that may not be ideal for those already suffering from trauma or disease. Another study conducted by Gonzalez et al. [23] also demonstrated the capability of an SSI to produce intelligible and natural speech using permanent magnetic articulography (PMA), but also suffers in usability due to the invasiveness of PMA and its current dependence on audio signals.

Using sEMG for alternative communication provides a noninvasive, easy-to-use alternative to EcoG- and PMA-based SSIs. Preliminary studies have shown the promise of sEMG-based SSIs to recognize a range of utterances including individual phonemes, isolated words, and even continuous speech with relatively high accuracy (e.g., [14,17,18,24,25]). Subsequent preliminary studies have begun to incorporate prosodic features in their sEMG-based SSI systems. By tracking articulatory muscle activity, sEMG-based SSIs from Johner et al. [26] and Vojtech et al. [18] were able to successfully distinguish emphasized words and questions from normal statements, demonstrating F1 scores of 0.68 and 0.92, respectively. While these studies demonstrated the ability of an sEMG-based SSI to detect prosodic features in speech, the metrics used may lack objectivity due to the large phonetic variation in how a word can be emphasized both within and across people [27]. As such, acoustic correlates of prosody could fulfill the unmet need to synthesize objective prosodic characteristics of speech more directly.

Past works have attempted to extract vocal pitch via estimates of fundamental frequency (*f*_*o*_) from sEMG activity but encountered difficulties without the use of machine learning methods. This is likely because voice production is primarily modulated by the intrinsic laryngeal muscles, which are not detectable using surface electrodes [28]. Instead, sEMG-based estimates of *f*_*o*_ have largely been attributed to changes in extrinsic laryngeal muscles. Due to the small, interdigitated, and overlapping nature of the extrinsic laryngeal musculature, however, it has been postulated that some muscles that are not involved in the control of voice *f*_*o*_ still contribute to the sEMG signal [29]. In turn, more recent work has turned to machine learning to disentangle voice *f*_*o*_ from sEMG signals. Nakamura et al. [30] was first to extract the *f*_*o*_ contour from an sEMG signal via Gaussian mixture model-based voice conversion. Diener et al. [31] improved on this work by quantizing the *f*_*o*_ values instead of estimating the contour from a trained model, and by introducing a feed-forward neural network for *f*_*o*_ estimation. However, both studies resulted in relatively low model performance between observed and predicted *f*_*o*_ estimates (*r* < 0.50). On top of low performance, these works also focused on pitch as a sole prosodic feature even though modulations in pitch, loudness, timing, and voice quality are often interdependent [32] (i.e., a syllable that is perceived as stressed is often produced with simultaneous increases in *f*_*o*_ and intensity; [33]). Nevertheless, these studies provide an important first step toward introducing linguistic prosody into synthetic speech for sEMG-based SSIs.

The aim of our current study was to investigate the efficacy of using sEMG to recognize and track continuous estimates of voice *f*_*o*_ and intensity. To achieve this goal, a series of time-, cepstral-, and frequency-domain features derived from sEMG signals was used to train deep regression models to estimate *f*_*o*_ and intensity of a concurrently recorded acoustic signal. Model performance in generating continuous estimates of *f*_*o*_ and intensity was characterized using outcome measures of percent error, correlation (via Pearson’s correlation and Lin’s concordance correlation), accuracy (via mean bias error), and precision (via root-mean-square error). We hypothesized that our regression models would demonstrate prediction errors below perceptible ranges reported in the literature for *f*_*o*_ (0.20–0.30 semitones; [34–36]) and intensity (2–5 dB SPL; [32,37]).

## 2. Materials and Methods

### 2.1. Participants

Ten adults with typical voices (5 female, 5 male; *M* = 29.8 years, *SD* = 9.6 years, *range*: 21–53 years) participated in the study. All participants were fluent in English and reported no history of voice, speech, language, or hearing disorders. One participant spoke English with an Arabic accent. All participants provided informed, written consent in compliance with the Western Institutional Review Board.

### 2.2. Experimental Protocol

**Figure 1.**
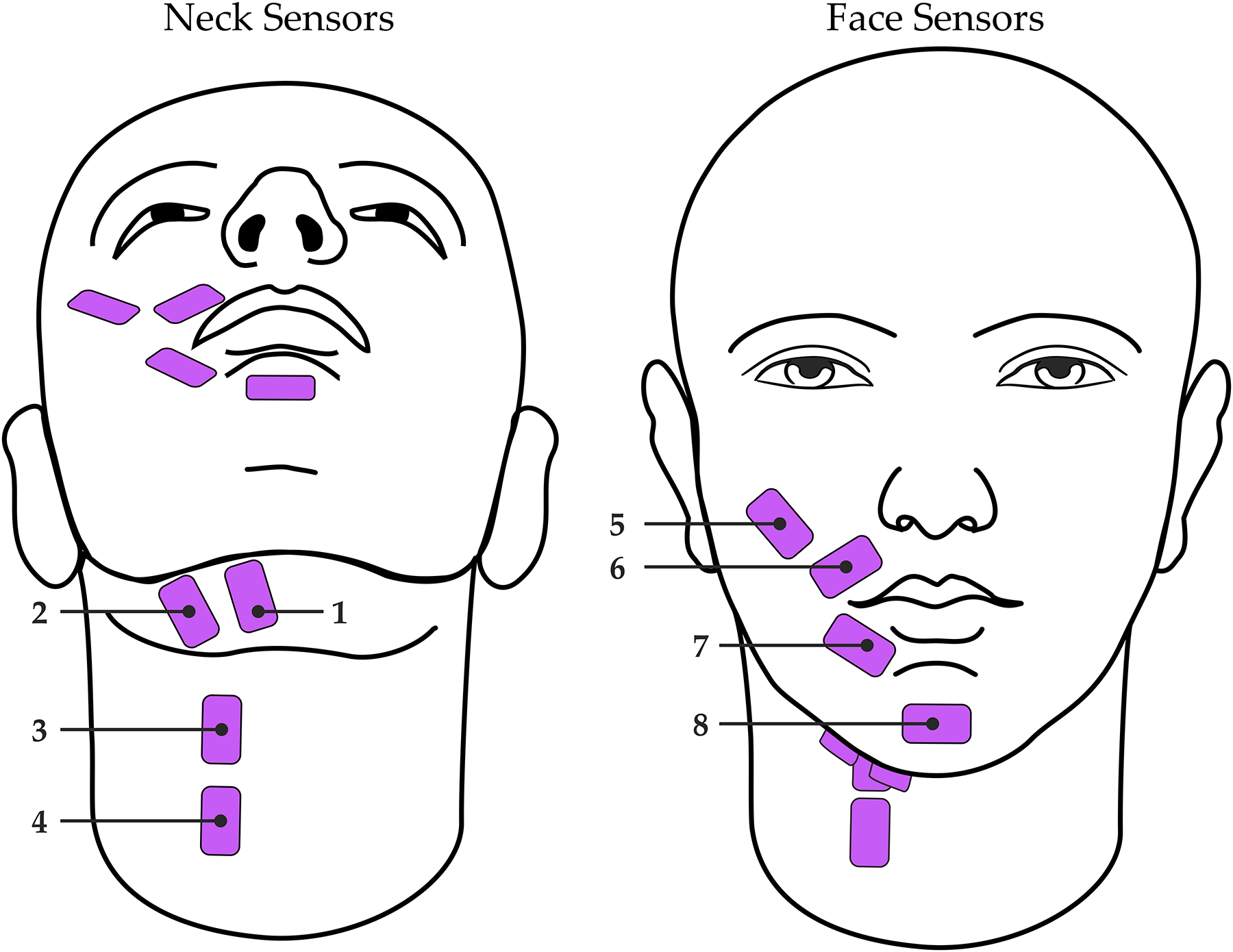
Configuration of sEMG sensors (pink) on the neck (**left**; sensors 1–4) and face (**right**; sensors 5–8).

Participants were seated throughout the study in a quiet room. Surface EMG signals were collected using eight single-differential electrode pairs connected to either of two wireless Trigno Quattro sensors (Delsys, Natick, MA, USA). Each differential electrode pair was placed over a distinct region of the face or neck as described in Meltzner et al. [14,15] (Figure 1). Neck sensor placements included the anterior belly of the digastric, mylohyoid, and geniohyoid (sensor 1; [38]); platysma, mylohyoid and stylohyoid (sensor 2; [38]); and platysma, thyrohyoid, omohyoid, and sternohyoid (sensors 3 and 4; [39]). Face sensor placements [40] included the zygomaticus major and/or minor, levator labii superioris, and levator anguli oris (sensor 5); orbicularis oris (sensors 6 and 7); and mentalis (sensor 8). Just prior to sensor adhesion, the surface of the skin was prepared via alcohol wipe and tape peel exfoliation methods to remove excess hair and skin oils [41,42]. The eight sensors were then adhered to the skin using double-sided, hypoallergenic tape. Signals were recorded at 2222 Hz, bandpass filtered with roll-off frequencies of 20 Hz and 450 Hz, and amplified by a gain of 300.

Acoustic signals were recorded using an omnidirectional microphone (Movo LV-6C XLR) instrumented to a headset; for each participant, the microphone was positioned 45° from the midline and 4–7 cm from the lips. Microphone signals were pre-amplified (ART Tube MP Project Series) and digitized at 44.1 kHz (National Instruments USB NI-6251).

Time-aligned acoustic and sEMG signal acquisition was managed through a triggering setup within Delsys EMGworks software and involved a custom trigger module to connect the National Instruments DAQ board and sEMG base station trigger port.

To calculate sound pressure level (dB SPL) for all voice recordings, electrolaryngeal pulses were played at the lips while a sound pressure level meter (Check Mate CM-140) measured dB SPL at the microphone. The known sound pressure levels were later used to calibrate the microphone recordings.

From here, participants produced seven different types of voice and speech data to collect a heterogenous sample of vocal activity. A detailed overview of the voice and speech tasks can be found in Appendix A, and are listed in brief below:

1. **Tones—**Sustained vowels /a/, /i/, /u/, and /ae/ produced for 3–5 s at a constant pitch and loudness (normative, high/low pitch, high/low loudness)
2. **Legatos—**Continuous slide from one pitch to another for vowels /a/, /i/, /u/, or /ae/
3. **VCV Syllables—**Vowel-consonant-vowel sequences (e.g., /afa/) where both vowels are equally stressed or only one vowel is stressed
4. **Phrases—**Standard, short speech tokens uttered in a normal speaking voice
5. **Reading Passages—**Standard passages uttered in a normal speaking voice
6. **Questions—**Short segments of unstructured speech in response to a question
7. **Monologues—**Long segments of unstructured speech in response to a prompt

Tasks were presented to participants on printouts displayed on a weighted-base copyholder (Fellowes 21128). Participants were instructed to notify the experimenter (authors J.V. or C.M.) when ready to begin a task; the experimenter would then start a recording to collect concurrent sEMG and acoustic data. In this way, participants proceeded through each task at their own pace. For tasks in which participants were instructed to alter their pitch and/or loudness (i.e., tones, legatos, nonsense words; see Appendix A), the degree of change was not assigned a specific sound pressure level or *f*_*o*_. Instead, it was determined by participants to fit within their comfortable conversational levels, similar to the recommended clinical instructions for instrumentally assessing voice [43]. An average of 2975.5 s of data was recorded for each participant (2501.9–3503.9 s), with recording duration by speech task shown in Table 1.

### 2.3. Data Processing

The sequence of data processing steps included: (1) signal alignment to align data recorded from the eight unique sEMG channels to the acoustic data recorded from the headset microphone, (2) voice *f*_*o*_ and intensity contour extraction, (3) feature extraction, and (4) data splitting. Each processing step is described in detail below.

#### 2.3.1. Signal Alignment

As each sEMG sensor was configured over distinct regions of the face or neck (with sensor configurations influenced by variable skin-electrode impedances and depth of the muscle from the skin surface, among other factors), a dynamic time warping (DTW) algorithm was implemented to capture the non-linear similarities between the acoustic data and the multiple, spatially distributed EMG sensors. For this procedure, the sEMG data from each sensor was first upsampled to 44.1 kHz to match the sampling rate of the acoustic data. An exact, memory-efficient algorithm for DTW was then employed using the *linmdtw* package [44] in Python (v.3.8) to compute signal alignments using a hop value of 0.010 s.

#### 2.3.2. Voice *f*_*o*_ and Intensity Contour Extraction

Two features were extracted from the acoustic data as outcome variables: voice *f*_*o*_ (Hz) and voice intensity (dB SPL). The *f*_*o*_ contour was extracted from each acoustic recording using the Praat autocorrelation-based algorithm [45] via the *Parselmouth* package [46] in Python. For this algorithm, minimum and maximum *f*_*o*_ values were set to 65 Hz and 475 Hz, respectively [47–49]. The time step for this algorithm was set to default (0.75/*minimum f*_*o*_).

The intensity contour was extracted following methods used in Praat, wherein the amplitude of a signal was first squared, then convolved with a Gaussian analysis window (Kaiser-20 with sidelobes below −190 dB). The duration of the analysis window was set to the default used in the Praat algorithm (3.2/*minimum f*_*o*_). Resulting intensity values were converted from units of dB to units of dB SPL using the known sound pressure levels acquired during data collection.

#### 2.3.3. Feature Extraction

Acoustic (*f*_*o*_ and intensity contours) and sEMG signals were windowed at a frame size of 40 ms with a 20-ms step shift for *f*_*o*_ data and 150 ms with a 30-ms step shift for intensity data. The *f*_*o*_ and intensity data were represented per frame by mean values. The sEMG data were represented per channel and per frame by a set of 20 common EMG features, which are listed in Table 2. All listed features were extracted for each of the 8 sEMG channels, then 24 redundant channel-features (e.g., the cross-correlation of channels 3 and 8 vs. the cross-correlation of channels 8 and 3) were removed. All features were then cascaded into a final vector with a dimension of 593 per sEMG sample.

Principal component analysis (PCA) was employed on the common set of 593 sEMG features from each participant to mitigate multicollinearity of features while constructing relevant features that capture most of the variance in the data. For each participant, the PCA criterion for the number of selected features was such that 90% of the variance in the data was explained [63–65]. This process yielded an average of 97.6 ± 2.1 features to characterize a given observation for intensity data and 106.0 ± 1.6 across participants for *f*_*o*_.

#### 2.3.4. Data Splitting

The amount of data available for model construction varied within and across participants due to differences in participant speech characteristics (e.g., speaking rate), task type (e.g., a sustained vowel vs. a long monologue), and outcome metric. For instance, there was substantially more data available for intensity than *f*_*o*_ since *f*_*o*_ could only be computed during voiced speech. Data splitting was therefore stratified across speech tasks to preserve the approximate proportions of the original dataset across models and to ensure an 80-20 (training-test) split.

Two methods were carried out to minimize overfitting: data augmentation and *k*-fold cross-validation. Data augmentation was applied as a regularization technique by injecting noise from a Gaussian distribution (based on the mean and standard deviations of the features) into the dataset [66,67]. Following, *k*-fold cross-validation with *k* = 5 folds was employed on the training data to quantify the variation in model performance [68]; resulting was a 60-20-20 split for training-validation-test sets.

### 2.4. Model Development

Model training was carried out using a Dell XPS 8950 desktop with the Windows 11 Pro 64-bit operating system. The processor was an Intel Core i7-12700 with 12 central processing unit cores. The computer was equipped with 32 GB random access memory, and the graphics processing unit of the computer was the NVIDIA GeForce RTX 3080.

Two types of *f*_*o*_ and intensity models were created: (1) single-speaker models, meaning that individual *f*_*o*_ and intensity models were trained for each participant, and (2) multi-speaker models, meaning that data from all 10 participants was used to train, validate, and test a single model for each outcome measure (*f*_*o*_, intensity). The former scheme was implemented to account for variations in the sEMG signal that may occur across participants due to differences in exact electrode configuration, skin-electrode impedances, skin and adipose thickness, and muscle activation during speech. The latter scheme was implemented to determine feasibility in creating a generalized architecture for estimating *f*_*o*_ and intensity in spite of person-specific variations in sEMG activity. Importantly, data augmentation was not implemented for the multi-speaker models due to the large amount of available data (spanning 10 participants).

**Figure 2.**
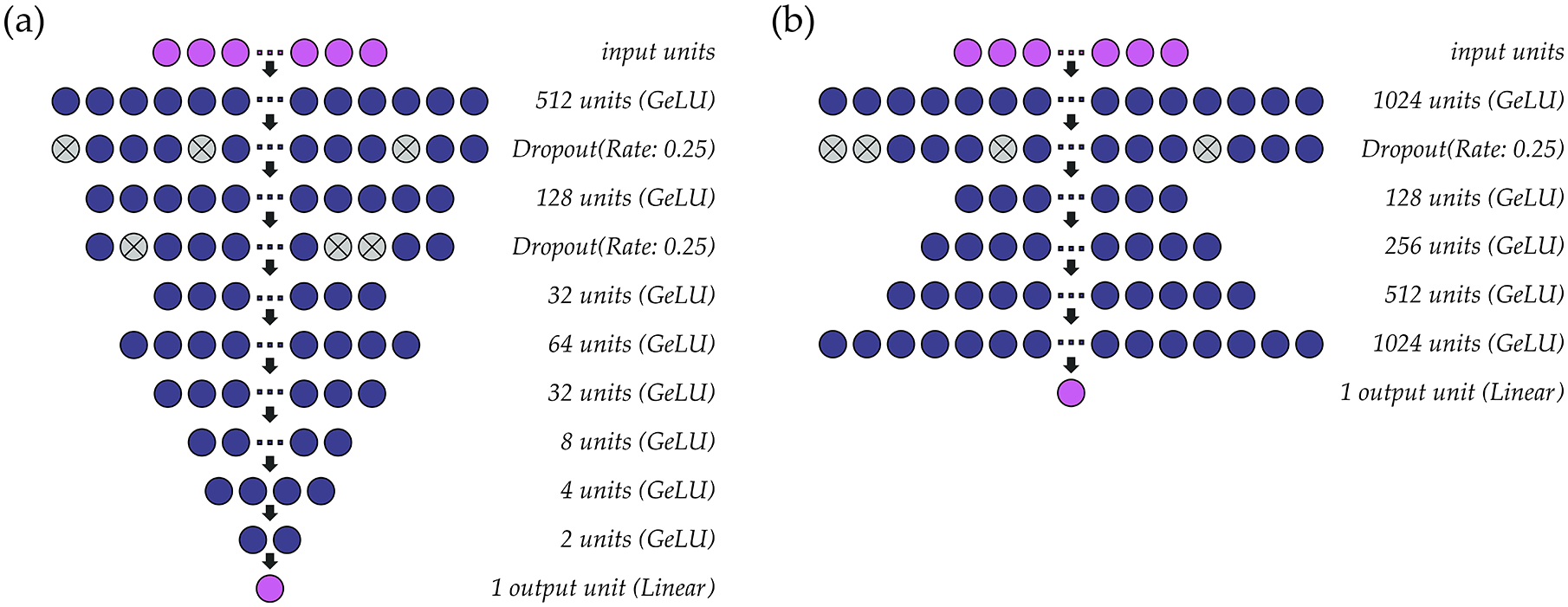
Structure of the single-speaker deep regression neural networks used to estimate (**a**) *f*_*o*_ and (**b**) intensity from sEMG signals.

A schematic representation of the single-speaker models for *f*_*o*_ and intensity can be found in Figure 2. The hidden layers within both models use the GeLU activation function. Parameter optimization for the *f*_*o*_ (Figure 2a) and intensity (Figure 2b) models is performed at a learning rate of 0.001 (batch size: 1024) and 0.005 (batch size: 2048), respectively, using the ADAM optimizer. As the models are intended to solve a regression problem, mean squared error is used as a loss function. Accordingly, the output layer for each model comprises one unit with a linear activation function. In the models for *f*_*o*_, all *f*_*o*_ values (predicted, observed) are standardized to semitones (ST) relative to a reference value based on the speaker’s average *f*_*o*_. Both models are deep regression neural networks that predict outcome values at a resolution of 0.01 ST (*f*_*o*_) or 0.01 dB SPL (intensity).

**Figure 3.**
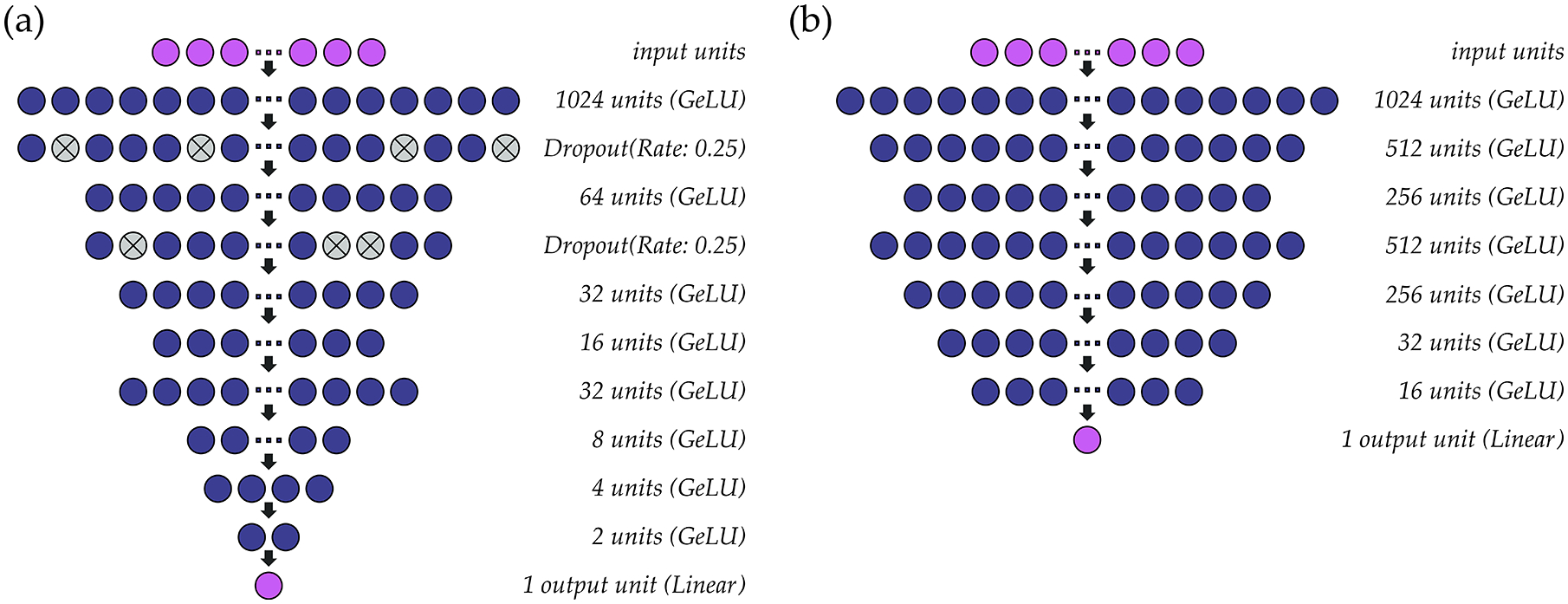
Structure of the multi-speaker deep regression neural networks used to estimate (**a**) *f*_*o*_ and (**b**) intensity from sEMG signals.

A schematic of the multi-speaker models that were constructed for *f*_*o*_ and intensity are shown in Figure 3. As in the single-speaker models, the hidden layers within both models use the GeLU activation function, mean squared error is used as a loss function, and the output layer consists of one unit with linear activation. Parameter optimization for *f*_*o*_ (Figure 3a) and intensity (Figure 3b) models is performed at a learning rate of 0.001 (batch size: 1024) and 0.0005 (batch size: 4096), respectively, using the ADAM optimizer. Batch normalization is included before the first activation layer of the intensity model to normalize the inputs to the first GeLU activation function. Due to differences in habitual pitch and loudness, *f*_*o*_ values are standardized to ST using a reference value of 90 Hz rather than the speaker’s average *f*_*o*_ and intensity values are normalized (0–1) within-participant across the available data. Both models are deep regression neural networks that predict outcome values at a resolution of 0.01 ST (*f*_*o*_) or 0.01 dB (intensity).

### 2.5. Model Performance

Model performance was quantified using metrics of mean absolute percent error (MAPE) as well as Pearson product-moment correlation coefficients (*r*) and Lin concordance correlation coefficients (CCC) to enable comparisons to the literature. Model performance was also quantified as the root-mean-square error (RMSE) and mean bias error (MBE) between observed and predicted estimates to provide insight into the precision and accuracy of *f*_*o*_ or intensity estimates. Performance for the training (60%) and validation (20%) data was compared across *k* = 5 folds. The fold that yielded the highest *CCC* value for validation data was identified as the final model for *f*_*o*_ or intensity. Final *f*_*o*_ and intensity models were then evaluated using the unseen test data (20%), and model performance was quantified per participant via MAPE, *r*, *CCC*, RMSE, and MBE.

## 3. Results

### 3.1. Single-Speaker Models

#### 3.1.1. Training and Validation Set Performance

Mean outcomes from both models (*f*_*o*_, intensity) were of the same magnitude between training and validation datasets, with validation results exhibiting slightly larger standard deviation values across the *k* = 5 cross-validation folds. Average model performance across cross-validation folds is shown by participant in Table B1 for *f*_*o*_ and Table B2 for intensity as well as summarized below.

Model performance in estimating *f*_*o*_ was comparable across cross-validation folds for training and validation datasets. Results for MAPE were, on average, 1.58% (*SD* = 0.24%) for the training data and 2.39% (*SD* = 0.72%) for the validation data. Findings were of similar magnitude for *r* and *CCC*, demonstrating average values of *r* = 0.98 (*SD* = 0.01) and *CCC* = 0.97 (*SD* = 0.01) for training data and *r* = 0.92 (*SD* = 0.05) and *CCC* = 0.92 (*SD* = 0.06) for validation data. Average training RMSE values were 0.34 ST (*SD* = 0.05 ST) and 0.52 ST (*SD* = 0.15 ST) for validation. Finally, MBE results were 0.27 ST (*SD* = 0.04 ST) and 0.41 ST (*SD* = 0.12 ST) for training and validation data, respectively.

Performance in estimating intensity demonstrated similar errors between training and validation datasets. Across the cross-validation folds, average training MAPE was 1.87% (*SD* = 0.41%) whereas validation MAPE was 3.31% (*SD* = 0.94%). Pearson’s *r* and Lin’s *CCC* values were above 0.90 for both datasets, averaging at *r* = 0.98 (*SD* = 0.01) and *CCC* = 0.98 (*SD* = 0.01) for training data with *r* = 0.92 (*SD* = 0.04) and *CCC* = 0.91 (*SD* = 0.04) for validation data. Average training RMSE was 2.38 dB SPL (*SD* = 0.96 dB SPL) whereas validation RMSE was 4.81 dB SPL (*SD* = 1.89 dB SPL). Results demonstrated an average MBE of 1.82 dB SPL (*SD* = 0.73 dB SPL) and 3.15 dB SPL (*SD* = 1.22 dB SPL) for training and validation data, respectively.

#### 3.1.2. Test Set Performance

**Figure 4.**
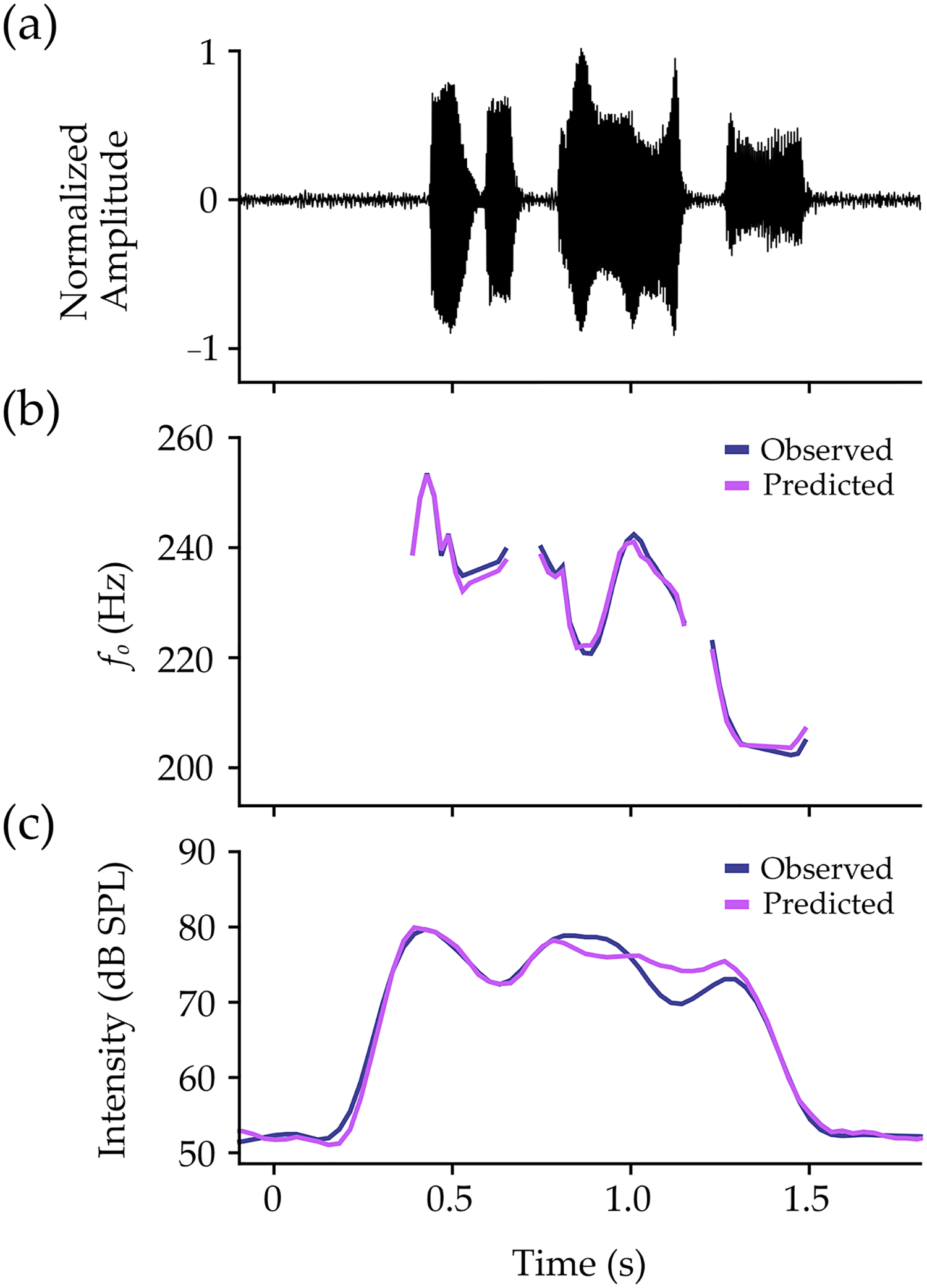
Example data for one participant from the phrase “Easy for you to say”. The normalized microphone signal is shown (**a**), with observed (navy lines) and predicted (pink lines) contours for (**b**) fo and (**c**) intensity. Contours for *f*_*o*_ have been converted from semitones to Hertz (Hz) for visualization purposes.

Within-participant performance on the test set is shown in Table 3. In the model for *f*_*o*_, MAPE was under 5% for all participants (*M* = 2.54%, *SD* = 0.72%). Pearson’s *r* and Lin’s *CCC* values demonstrated mean values of *r* = 0.92 (*SD* = 0.05) and *CCC* = 0.91 (*SD* = 0.07). The mean ST error between observed and predicted values was 0.01 ST (*SD* = 0.08 ST), with precision estimates averaging at 0.56 ST (*SD* = 0.16 ST). An example of observed and predicted contours is shown for *f*_*o*_ in Figure 4b.

Results for intensity also showed MAPE values under 5% for all participants (*M* = 2.38%, *SD* = 0.97%). Pearson’s *r* and Lin’s *CCC* values were over 0.94 for all participants, showing mean values of *r* = 0.97 (*SD* = 0.01) and *CCC* = 0.97 (*SD* = 0.01). The RMSE between observed and predicted values was 3.25 dB SPL (*SD* = 1.18 dB SPL), with MBE averaging at 0.21 dB SPL (*SD* = 0.85 dB SPL). An example of observed and predicted contours is shown for intensity in Figure 4c.

### 3.2. Multi-Speaker Models

Results for the multi-speaker *f*_*o*_ model is shown for the training, validation, and test datasets in Table 4. The multi-speaker *f*_*o*_ model demonstrated similar trends across outcome metrics, wherein performance was worst on the validation data, followed by the training data. Performance in the test set was comparable to the training and validation data. Specifically, MBE which was lowest (most accurate) for the test dataset (0.13 ST). Average MAPE values were below 10% across all three dataset types, with poor validation correlations (*r* = 0.25, *CCC* = 0.10) and moderate training (*r* = 0.41, *CCC* = 0.17) and test (*r* = 0.36, *CCC* = 0.25) correlations.

Results for the multi-speaker intensity model is shown for the training, validation, and test datasets in Table 5. As the multi-speaker model was evaluated on normalized SPL values, results for RMSE and MBE are shown in units of decibels (dB). The multi-speaker intensity model showed the best performance on the test dataset in terms of correlation (*r* = 0.56, *CCC* = 0.48) and accuracy (−0.02 dB). MAPE was under 15% for all datasets, with poor-to-moderate training (*r* = 0.51, *CCC* = 0.44) and validation (*r* = 0.32, *CCC* = 0.24) correlations. Finally, the precision of intensity estimates was comparable across the three datasets (0.11–0.12 dB).

## 4. Discussion

The goal of this study was to determine the feasibility of using sEMG signals of the face and neck to predict two primary attributes of linguistic prosody: voice *f*_*o*_ and intensity. This study builds on our primary work in using sEMG activity for silent speech recognition (i.e., identifying the words in a message; [14,15]) and for classifying basic manipulations in prosody (i.e., identifying how the words in a message are conveyed; [18]). Taking this past work into account, the current study successfully demonstrates efficacy in using sEMG as an alternative method for detecting prosody via continuous estimates of *f*_*o*_ and intensity.

### 4.1. Single-Speaker vs. Multi-Speaker Models

Single- and multi-speaker models were examined in this work. The single-speaker models were trained and tested on data recorded for an individual participant, whereas the multi-speaker models were trained and tested from the data of 10 participants. The motivation for examining both single- and multi-speaker models stems from the reliance of each model on the acoustic signal. Both models rely on audio data for training, but the multi-speaker models could, in theory, be used by other individuals without an inherent reliance on their specific audio data. Applications for this latter model include situations in which the individual cannot supply acoustic data to train a model (e.g., those who cannot voice due to trauma or disease, such as laryngectomees).

Unsurprisingly, our single-speaker models performed better than the multi-speaker counterparts, as sEMG signals are speaker-dependent due to skin-electrode impedances, skin and adipose thickness, as well as differences in muscle activation during speech. Indeed, most prior works in this area focus on single-speaker models for this very reason (e.g., [18,25,31,69]). We argue that the overall performance of the multi-speaker models is still promising, as our results provide preliminary evidence of predicting *f*_*o*_ and intensity within 10% and 15% error, respectively. Additional work is still necessary to extend this approach toward a robust system that is independent of the user’s acoustic information. Moreover, the multi-speaker models examined here included data from all 10 participants with each dataset (training, validation, test), such that model performance on unseen participants was not evaluated. This was done to determine the feasibility of using a single model to capture sEMG and acoustic variability across individuals to estimate *f*_*o*_ or intensity prior to doing so in unseen individuals. However, future work should aim to train and test such models on independent participants to determine the generalizability of our approach (e.g., for those who cannot contribute acoustic information to model training). Future work should also consider acquiring more data from individuals across a wide range of vocal function as one potential method of increasing the generalizability of our multi-speaker models, as a small sample size of only ten individuals with typical voices was included here.

### 4.2. Significance of Single-Speaker Model Performance

#### 4.2.1. Comparisons to Model Performance in the Literature

We investigated the ability of deep regression models to predict discrete estimates of voice *f*_*o*_ and intensity from sEMG data of the face and neck musculature. This work expands on studies from the literature that utilize different machine learning approaches for estimating prosodic information from EMG data alone. Our results notably surpass values reported in the literature for *f*_*o*_ estimation while also detailing one of the first accounts (to our knowledge) of predicting vocal intensity (loudness) from sEMG signals.

The use of sEMG for estimating voice *f*_*o*_ is a concept that has been scarcely explored over the past decade, resulting in a limited number of comparative works. A pioneering study by Nakamura et al. [30] sought to use a Gaussian mixture model-based approach to estimate *f*_*o*_ from five sEMG sensors, demonstrating an average correlation between observed and predicted *f*_*o*_ values of *r* = 0.49 across three speakers. De Armas et al. [69] sought to predict *f*_*o*_ using support vector machine regression and classification from sEMG traces. In estimating *f*_*o*_ from tones, the authors reported an average correlation of *r* = 0.96; however, this correlation decreased to *r* = 0.88 when estimating *f*_*o*_ from phrases. Making use of a similar protocol, Ahmadi et al. [70] aimed to achieve better correlations in predicting *f*_*o*_ values from sEMG data as compared to De Armas et al. [69]. As anticipated, the authors reported an average correlation of *r* = 0.93 when estimating *f*_*o*_ from phrases from a small sample of three participants.

Although the average correlations in Nakamura et al. [30], De Armas et al. [69], and Ahmadi et al. [70] are lower than or comparable to those observed in the current study (*r* = 0.92), it must be noted that it is difficult to directly compare model performance across studies. There are substantial differences in methodology across these works, ranging from experimental setup (e.g., sEMG hardware), protocol (e.g., vocal tasks), and model construction (e.g., support vector machine vs. deep regression models) that complicate interpretations for why a given model may have performed better than another. For instance, our study utilized bipolar sEMG sensors sampled at 2222 Hz whereas that of Nakamura et al. [30] acquired sEMG activity via a mix of bipolar and monopolar sEMG sensors sampled at 600 Hz. Nakamura et al. [30] recorded participants as they produced phrases and De Armas et al. [69] and Ahmadi et al. [70] recorded participants as they produced tones, legatos, and phrases, whereas the current study incorporated these three vocal tasks as well as additional types of continuous (i.e., reading passages) and spontaneous (i.e., monologues and questions) speech. Thus, we caution readers to consider the differences in methodology across sEMG-based SSI studies rather than taking the correlative results presented here at face value.

Still, it must be considered that developing an SSI that estimates *f*_*o*_ from basic speech units like tones or legatos may be a necessary first step to demonstrate the proof of principle; however, the introduction of continuous and spontaneous speech tasks as in the current study is important to consider for ensuring ecological validity. In fact, these tasks represented more than 52% of the total data recorded in the study. Without such tasks, the SSI is inherently constrained in requiring basic *f*_*o*_ manipulations (in the case of tones or legatos) and pauses (in the case of phrases) to decipher *f*_*o*_. Moreover, De Armas et al. [69] observed an average RMSE of 2.81 ST for *f*_*o*_ estimation, which is about 5-fold greater than the average RMSE obtained in the current work of 0.56 ST. These results show the importance of using multiple outcome metrics to provide comprehensive insight into model performance.

More recently, Diener et al. [31] examined the relationship between acoustic (observed) and sEMG-derived (predicted) speech features when using electrode arrays. The authors opted to build upon their prior work by deriving “quantized” estimates of *f*_*o*_ rather than continuous estimates; however, the authors still observed poor correlative performance (*r* = 0.27). A shift from direct *f*_*o*_ estimation can be observed in Janke et al. [69] and Botelho et al. [70], wherein algorithmic performance did not specifically include *f*_*o*_ as an outcome. Instead, the authors sought to determine the output quality of the speech (via mel-cepstral distortion and mel-frequency cepstral coefficients) rather than the quality of specific prosodic attributes (e.g., *f*_*o*_, intensity). Though outside the scope of the current study, future work could incorporate these speech quality features in addition to the prosodic features examined here.

#### 4.2.2. Comparisons to Meaningful Changes in *f*_*o*_ and Intensity

Our results show a high degree of agreement between acoustic and sEMG-derived estimates of *f*_*o*_ and intensity within each participant. Within this analysis, RMSE and MBE were calculated as an estimate of prediction precision and accuracy, respectively. For multi-speaker *f*_*o*_ models, our results indicate a mean MBE of 0.03 ST. This suggests that our models will, on average, generate a positively biased systematic error (i.e., overestimated) of approximately 0.03 ST. The average RMSE across participants was 0.56 ST, indicating that the average spread of errors will approach 0.56 ST when using our models to estimate *f*_*o*_. For single-speaker intensity models, our findings indicate an average MBE of 0.21 dB SPL and RMSE of 3.25 dB SPL. These results suggest that using our models to estimate intensity from sEMG signals will generate a positively biased error of 0.21 dB SPL, with the precision of intensity estimates approaching 3.25 dB SPL.

It is important to consider how these errors between observed and predicted *f*_*o*_ values compare to meaningful differences in the literature. For instance, the average vocal pitch discrimination ability of an adult has been reported to be within the range of 0.20 to 0.30 ST [34–36]. The average accuracy of our *f*_*o*_ estimations was found to be 0.01 ST, meaning that the MBE associated with using our single-speaker *f*_*o*_ models is on the order of one magnitude smaller than the pitch discrimination abilities of a typical adult reported in the literature. This suggests that erroneous *f*_*o*_ values predicted by our model will, on average, not be perceived by the typical adult.

The average errors obtained for vocal intensity can also be compared to meaningful values reported in the literature. Specifically, the mean short-term variation in vocal intensity has been reported to be approximately 2–5 dB SPL for adults [37,71]. With an average MBE of 0.21 dB SPL, our results suggest that average erroneous intensity estimates predicted by the single-speaker intensity models will be within the bounds of typical, short-term variations in vocal intensity.

### 4.3. Physiological Interprations of Model Performance

The results of the current study suggest that *f*_*o*_ and intensity can be sufficiently estimated on a per-individual basis from sEMG activity of the face and neck. The notion that these prosodic attributes—*f*_*o*_, in particular—can be estimated from relatively surface-level muscles is interesting when considering the orofacial and laryngeal muscles necessary for voicing, as voice production is primarily modulated by the intrinsic laryngeal muscles. Specifically, the primary function of the cricothyroid is to lengthen and stretch the vocal folds to, in turn, increase the vibratory rate of the vocal folds (and thus, increase *f*_*o*_; [72]). The thyroarytenoid, on the other hand, stabilizes the onset of phonation and contributes to increases in the vibratory rate of the vocal folds [71,73]. Taken together, the contraction force of these muscles has been shown to jointly increase with increases in voice *f*_*o*_ and intensity [74].

Due to the relatively deep location of muscles within the larynx, however, it is unlikely that the activity of the cricothyroid or thyroarytenoid contributes to the detected signal when using surface electrodes [75]. Instead, it is more likely that activity from the extrinsic laryngeal muscles—which induce changes in laryngeal elevation to *indirectly* affect the vibratory rate of the vocal folds [76]—along with muscles of the face contributed to the detected sEMG signals. Indeed, prior work examining the thyrohyoid, sternothyroid, and sternohyoid (“strap muscles”) during different vocal tasks suggests that these extrinsic laryngeal muscles are involved in the dynamic modulation of voice production (i.e., rising or falling frequency) rather than in the specific *f*_*o*_ itself [77]. It has also been reported that the strap muscles are differentially active during high and low *f*_*o*_ productions [78–80], as well as during voice productions at varying loudness levels [81]. In addition to the extrinsic laryngeal muscles, changes in vocal intensity from habitual loudness to either softer or louder levels has been shown to significantly alter average sEMG amplitude of the lip muscles [82]. Increases in voice *f*_*o*_ have also been associated with differential changes in surface electromyographic activity of the face [83].

Taking these prior works into account, it is likely that our models were able to learn from the sEMG activity from the sensors placed over the extrinsic laryngeal muscles (i.e., sensors 1–4 in Figure 1) and the orofacial muscles (i.e., sensors 5–8 in Figure 1) to understand how a given participant’s dynamic patterns used to modulate their voice, including *f*_*o*_ and intensity. It is also important to note that these past studies examined the amplitude of the sEMG signal relative to voice *f*_*o*_ and intensity, whereas the current study leveraged a combination of 57 time-, frequency-, and cepstral-domain features from the sEMG signal. Our results suggest that this combination of features can effectively detect changes in extrinsic laryngeal and orofacial muscle activity in a way that is associated with changes in voice *f*_*o*_ and intensity. Additional investigations should be undertaken to examine these voice attributes relative to specific sEMG sensor sites (e.g., over the strap muscles vs. over the lip muscles) to further elucidate the relationship between extrinsic laryngeal or orofacial muscle activity and *f*_*o*_ or intensity.

### 4.4. Limitations and Future Directions

Although the current study details favorable results regarding the performance of deep regression neural networks for predicting voice *f*_*o*_ and intensity, further investigation is warranted to continue to enhance the accuracy and accessibility of the models. For instance, voice *f*_*o*_ is relatively position-independent whereas voice intensity may vary based on the distance from the microphone to the mouth. Though outside the scope of this study—which sought to demonstrate the proof-of-concept that *f*_*o*_ and intensity could be estimated from sEMG activity of the face and neck—future work should investigate normalization methods to account for differences in microphone distance that may occur within and across individuals who use the system. Within this vein, our multi-speaker models did not perform as well as single-speaker models for *f*_*o*_ and intensity predictions. As a result, the current methods must rely on an individual’s acoustic signal to train a model, hampering usability in the target population of individuals who are unable to voice (due to trauma or disease). As discussed in Section 4.2, future work is needed to increase the accuracy and precision of multi-speaker *f*_*o*_ and intensity models possibly by expanding the number of participants as is done for acoustic speech recognition models (e.g., [84–86]); in this way, the models could be trained using sEMG and acoustic data from individuals with typical voices and then tested (used) by those without a voice.

Voice *f*_*o*_ and intensity are important as suprasegmental characteristics of speech but are not the only two attributes of linguistic prosody. Though outside the scope of the current study, future investigations should incorporate attributes of timing (e.g., word duration) and voice quality into the models for *f*_*o*_ and intensity estimation. Within a similar vein, the current study aimed to examine suprasegmental characteristics of speech separately from segmental characteristics, such as word or phoneme prediction. Subsequent efforts will be undertaken to combine our approach with the word recognition methods detailed in our prior works toward developing a prosodic, sEMG-based SSI.

## 5. Conclusions

Surface EMG is a promising modality for SSIs due to its noninvasive nature and ease of application; however, most sEMG-based SSIs fail to convey the expressive attributes of prosody, including pitch and loudness. This work details the construction and evaluation of deep regression neural networks for predicting continuous estimates of voice *f*_*o*_ and intensity from sEMG recordings from muscles of the face and neck. When evaluated in ten participants, model estimation of *f*_*o*_ yielded an average accuracy of 0.01 ST and precision of 0.56 ST while model estimation of intensity provided a mean accuracy of 0.21 dB SPL and precision of 3.25 dB SPL. The average accuracy of *f*_*o*_ estimation was approximately one order of magnitude smaller than the pitch discrimination abilities of a typical adult, suggesting that erroneous *f*_*o*_ values predicted by our model will, on average, not be perceived by the typical adult. Moreover, our results suggest that erroneous model estimates of intensity will, on average, be within the bounds of typical, short-term variations in vocal intensity. This study is a critical first step toward introducing linguistic prosody into synthetic speech for sEMG-based SSIs.

## Figures and Tables

**Table 1.** Recording duration by speech task, shown as mean (range).

Speech Task	Recording Duration (sec)
Tones	351.7 (232.2–620.7)
Legatos	132.1 (97.4–205.8)
VCV Syllables	284.4 (174.6–464.0)
Phrases	649.8 (523.5–790.9)
Reading Passages	1041.1 (888.9–1209.0)
Questions	241.6 (168.5–330.8)
Monologues	274.8 (214.5–374.9)

**Table 2.** Features used in sEMG data processing.

	Feature	Dimension per Channel	References
1	Beta coherence	8	[48, 49]
2	Central frequency variance	1	[50, 51]
3	Coherence	8	[48, 49]
4	Cross-correlation	8	[52]
5	Daubechies 2 wavelet coefficients, maximum (peak)	4	[53]
6	Daubechies 2 wavelet coefficients, mean	4	[53]
7	Daubechies 2 wavelet coefficients, variance	4	[53]
8	Maximum (peak) frequency	1	[50, 53]
9	Mean absolute value	1	[54–59]
10	Mean frequency	1	[57, 58]
11	Mean power density	1	[51, 60]
12	Median frequency	1	[58, 59]
13	Mel-frequency cepstral coefficients	24	[14, 15, 18]
14	Power density wavelength	1	[55]
15	Root mean square	1	[54, 55, 58, 59]
16	Slope sign change	1	[55, 58]
17	Spectral moments	3	[50, 55, 59, 60]
18	Variance	1	[54, 55, 58, 59]
19	Waveform length	1	[55, 57–59]
20	Zero crossings	1	[17, 55, 57, 58]

**Table 3.** Single-dependent *f*_*o*_ and intensity model performance on the test set for 10 participants.

ID	*f*_*o*_					Intensity				
	MAPE (%)	*r*	*CCC*	RMSE (ST)	MBE (ST)	MAPE (%)	*r*	*CCC*	RMSE (dB SPL)	MBE (db SPL)
1	1.75	0.96	0.95	0.38	0.05	2.21	0.98	0.98	2.06	−0.27
2	1.82	0.95	0.94	0.40	0.09	2.44	0.98	0.98	2.23	−0.03
3	2.49	0.94	0.94	0.55	−0.02	1.60	0.97	0.97	4.17	0.88
4	2.33	0.95	0.94	0.51	0.03	1.46	0.98	0.98	3.53	−0.69
5	2.36	0.94	0.94	0.52	−0.04	3.40	0.96	0.95	3.35	0.85
6	2.26	0.96	0.95	0.50	−0.01	1.36	0.97	0.97	3.33	−0.35
7	2.25	0.96	0.96	0.49	0.00	4.60	0.94	0.94	6.17	2.04
8	3.79	0.79	0.74	0.86	−0.16	2.75	0.98	0.98	2.41	0.10
9	2.41	0.95	0.94	0.53	0.07	1.54	0.97	0.97	3.11	−1.00
10	4.03	0.83	0.80	0.90	0.12	2.50	0.98	0.98	2.17	0.52

**Table 4.** Subject-independent *f*_*o*_ model performance on training, validation, and test datasets.

Dataset	MAPE (%)	*r*	*CCC*	RMSE (ST)	MBE (ST)
Training*	8.15 (0.53)	0.41 (0.04)	0.17 (0.12)	1.67 (0.12)	1.42 (0.11)
Validation*	8.16 (0.62)	0.25 (0.10)	0.10 (0.07)	1.66 (0.11)	1.42 (0.11)
Test	7.95	0.36	0.25	1.65	0.13

* Results are shown across cross-validation folds as mean (standard deviation) for training and validation datasets.

**Table 5.** Subject-independent intensity model performance on training, validation, and test datasets.

Dataset	MAPE (%)	*r*	*CCC*	RMSE (dB)	MBE (dB)
Training*	12.80 (0.49)	0.41 (0.05)	0.30 (0.05)	0.11 (0.01)	0.09 (0.01)
Validation*	13.79 (1.57)	0.24 (0.06)	0.20 (0.05)	0.13 (0.01)	0.10 (0.01)
Test	13.94	0.52	0.42	0.12	−0.04

* Results are shown across cross-validation folds as mean (standard deviation) for training and validation datasets.

## Data Availability

The data presented in this study are available on request from the corresponding author. The data are not publicly available due to the identifiable nature of voice acoustic recordings.

## Appendix A

**Table A1.** Overview of speech tasks.

Task	Description	Subtasks
Tones	Sustained vowels /a/, /i/, /u/, and /ae/ produced at a constant pitch and loudness, repeated three times for each variation	1. Typical pitch and loudness
		2. High pitch
		3. Low pitch
		4. High intensity
		5. Low intensity
Legatos	Continuous slide from one pitch to another using the vowels /a/, /i/, /u/, and /ae/	1. Low pitch
		2. Mid pitch
		3. High pitch
VCV^a^ Syllables	Bisyllabic productions repeated three times for each variation	1. Equal stress
		2. Stress on first vowel
		3. Stress on second vowel
Phrases	Standard, short speech tokens that introduce various stress placements	1. UNL Phrases
		2. RFF Phrases
Reading Passages	Standard reading passages that introduce various stress placements	1. The Caterpillar Passage
		2. My Grandfather Passage
		3. Rainbow Passage
		4. Golf Passage
		5. Pronunciation Reading Passage
		6. Please Call Stella
		7. Comma Gets a Cure
		8. Frog and Toad
		9. Excerpt from Harry Potter and the Chamber of Secrets
		10. Excerpt from The Little Prince
		11. Excerpt from the Boston University Radio Speech Corpus
Questions	Short (<30 seconds) segment of unstructured, conversational speech	1. If you could live in any decade, what would it be and why?
		2. What is your favorite time of day and why?
		3. If you were to make a movie about your life, what genre would you choose and why?
		4. How did you get here today?
		5. Do you have any vacation or travel plans?
		6. Tell me about how the weather has been recently.
		7. What did you do last weekend?
Monologues	Long (>60 seconds) segment of unstructured, conversational speech	1. Tell me how to make a peanut butter and jelly sandwich.
		2. Tell me how you do your laundry.
		3. Tell me how you get ready for work.
		4. Tell me how you make your bed.

a VCV = vowel-consonant-vowel, with vowels /a/, /i/, or /u/ and consonants /f/, /v/, /p/, or /b/

## Appendix B

**Table B1.** Performance for the *f*_*o*_ models across *k* = 5 cross-validation training (Train) and validation (Valid) datasets for N = 10 participants. Results are shown as mean(standard deviation).

ID	MAPE (%)		*r*		*CCC*		RMSE (ST)		MBE (ST)	
	Train	*Valid*	Train	*Valid*	Train	*Valid*	Train	*Valid*	Train	*Valid*
1	1.36(0.04)	*2.21(0.71)*	0.98(0.00)	*0.92(0.06)*	0.97(0.00)	*0.90(0.08)*	0.29(0.01)	*0.49(0.16)*	0.23(0.01)	*0.38(0.13)*
2	1.27(0.03)	*2.07(0.43)*	0.98(0.00)	*0.93(0.04)*	0.97(0.00)	*0.92(0.04)*	0.28(0.01)	*0.46(0.10)*	0.22(0.01)	*0.36(0.07)*
3	1.60(0.02)	*1.69(0.08)*	0.98(0.00)	*0.98(0.00)*	0.98(0.00)	*0.97(0.00)*	0.34(0.00)	*0.36(0.02)*	0.28(0.00)	*0.29(0.01)*
4	1.79(0.04)	*2.69(0.75)*	0.97(0.00)	*0.92(0.06)*	0.97(0.00)	*0.91(0.06)*	0.38(0.01)	*0.60(0.18)*	0.31(0.01)	*0.47(0.13)*
5	1.66(0.02)	*3.67(2.63)*	0.97(0.00)	*0.80(0.29)*	0.97(0.00)	*0.78(0.32)*	0.36(0.01)	*0.77(0.51)*	0.29(0.00)	*0.62(0.43)*
6	1.79(0.02)	*2.77(0.80)*	0.97(0.00)	*0.93(0.06)*	0.97(0.00)	*0.92(0.06)*	0.39(0.01)	*0.61(0.20)*	0.31(0.00)	*0.48(0.14)*
7	1.63(0.02)	*2.66(0.84)*	0.98(0.00)	*0.93(0.06)*	0.98(0.00)	*0.92(0.06)*	0.35(0.00)	*0.59(0.20)*	0.28(0.00)	*0.46(0.15)*
8	1.13(0.01)	*1.23(0.09)*	0.98(0.00)	*0.98(0.00)*	0.98(0.00)	*0.98(0.00)*	0.25(0.00)	*0.27(0.02)*	0.19(0.00)	*0.21(0.02)*
9	1.80(0.03)	*3.03(1.18)*	0.97(0.00)	*0.89(0.11)*	0.97(0.00)	*0.88(0.13)*	0.38(0.01)	*0.68(0.29)*	0.31(0.00)	*0.53(0.21)*
10	1.78(0.01)	*1.86(0.02)*	0.97(0.00)	*0.97(0.00)*	0.97(0.00)	*0.97(0.00)*	0.38(0.00)	*0.40(0.00)*	0.31(0.00)	*0.32(0.00)*

**Table B2.** Performance for the intensity models across *k* = 5 cross-validation training (Train) and validation (Valid) datasets for N = 10 participants. Results are shown as mean(standard deviation).

ID	MAPE (%)		*r*		*CCC*		RMSE (dB SPL)		MBE (dB SPL)	
	Train	*Valid*	Train	*Valid*	Train	*Valid*	Train	*Valid*	Train	*Valid*
1	1.96(0.31)	*3.88(2.77)*	0.99(0.00)	*0.90(0.15)*	0.99(0.00)	*0.89(0.17)*	1.7(0.25)	*3.94(3.00)*	1.28(0.21)	*2.60(1.95)*
2	2.30(1.01)	*3.80(1.58)*	0.99(0.00)	*0.95(0.05)*	0.99(0.01)	*0.94(0.05)*	1.72(0.67)	*3.33(1.38)*	1.36(0.59)	*2.28(0.93)*
3	1.68(0.36)	*3.44(2.13)*	0.97(0.01)	*0.81(0.23)*	0.97(0.01)	*0.81(0.24)*	3.90(0.88)	*9.30(6.42)*	2.94(0.62)	*5.96(3.70)*
4	1.78(0.81)	*2.39(0.80)*	0.96(0.03)	*0.93(0.05)*	0.96(0.04)	*0.92(0.05)*	4.16(1.85)	*5.91(2.01)*	3.20(1.41)	*4.31(1.41)*
5	2.11(0.16)	*4.27(1.75)*	0.99(0.00)	*0.92(0.08)*	0.99(0.00)	*0.91(0.08)*	1.68(0.09)	*4.21(1.99)*	1.29(0.09)	*2.67(1.15)*
6	1.27(0.16)	*2.07(0.64)*	0.98(0.01)	*0.93(0.06)*	0.98(0.01)	*0.92(0.07)*	2.83(0.35)	*5.06(1.91)*	2.17(0.27)	*3.54(1.12)*
7	2.46(0.09)	*4.65(0.96)*	0.99(0.00)	*0.94(0.03)*	0.99(0.00)	*0.94(0.03)*	2.33(0.09)	*5.87(1.47)*	1.75(0.08)	*3.41(0.72)*
8	2.11(0.12)	*3.55(1.50)*	0.99(0.00)	*0.95(0.06)*	0.99(0.00)	*0.94(0.06)*	1.72(0.08)	*3.25(1.51)*	1.32(0.07)	*2.21(0.91)*
9	1.20(0.21)	*1.83(0.84)*	0.98(0.01)	*0.91(0.12)*	0.98(0.01)	*0.91(0.12)*	2.28(0.46)	*4.39(3.00)*	1.74(0.30)	*2.62(1.18)*
10	1.88(0.05)	*3.18(1.29)*	0.99(0.00)	*0.96(0.04)*	0.99(0.00)	*0.96(0.04)*	1.50(0.03)	*2.88(1.34)*	1.15(0.03)	*1.93(0.76)*
